# Tuberculosis

**Published:** 1904-06-11

**Authors:** 


### TUBERCULOSIS.

**Treatment.**—All advocates of sanatorium treatment call attention to the fact that though existing disease may be cured, reinfection is possible. The figures quoted by Williams [1] from German and Swiss sanatoria clearly show this. The German statistics, drawn from 6,000 cases, show that of 68 per cent. discharged as capable of work in 1898, only 27 per cent. remained so at the end of 1901; at the Basel Davos Sanatorium, of 70·1 per cent. discharged at the end of 1899, 58·2 per cent. remained well and capable at the end of 1901. The importance of submitting patients early to this treatment is shown by the fact that from 2,954 Swiss cases 83·9 per cent. were discharged with unimpaired work-power from among early cases, and only 10·1 per cent. from among those in the later stages. As to construction, Williams points out that wooden huts have disadvantages as regards service, and are very liable to destruction by fire; he urges the importance of plain but durable structures, though in America huts are used in the Adirondacks, and in Germany the Docker huts find favour. Similar structures are used at Parkestone, near Bournemouth. In New York good results are reported from tent life for the tuberculous insane (Robinson[2]). Williams advocates the erection of shelters or Liegenhallen facing north for summer and south for winter, an air space of at least 1,000 cubic feet per head in the bedrooms, which should be warmed by radiators and not by open fires. He insists on the importance of carefully graduated exercise, and points out the fallacy of regarding increase of weight under excessive feeding as an index of improvement. It only means an increase in fat, and may, through increased blood pressure, lead to fatal hæmoptysis. Sun baths he thinks have done good in some cases, but are not always practicable in this country. Gordon[3] has shown the effect of wet westerly winds in raising the mortality from phthisis, and Loomis[4] points out that though altitude is somewhat disregarded on the Continent, the two most successful sanatoria are built at high elevations above the sea, and Williams attributes the better results in Swiss sanatoria to climate and altitude. Folks[5] notes that in the sanatorium at Manhattan the discontinuance of cod-liver oil and alcohol in any large quantity had not been found detrimental to the patients. Direct curative agents are still being sought for as a means of dealing with advanced cases, and in others of shortening the time necessarily spent at sanatoria. Marmorek[6] has prepared a serum by growing tubercle bacilli on a special medium which eliminates the production of tuberculin but causes another product to appear, toxic for small animals, but not producing a reaction in those attacked by tuberculosis. He claims good results in cases of tuberculous pleurisy (six cases out of seven cured), some amelioration in phthisis but no effect in tuberculous meningitis. Dieulafoy[7] reports on seven cases treated by this serum: four pulmonary, two pulmonary and laryngeal, and one pleural with effusion, five died, one with fibroid changes in the lungs and one with pleurisy are still alive. In guinea-pigs experimentally infected no good results were obtained. Le Dentu[8] treated one case of pulmonary and bone tuberculosis, and Hallopeau,[9] seven cases of tuberculosis of the skin quite unsuccessfully. Monod[10] claimed good results in local or glandular tuberculosis, but Championière[11] obtained no improvement in five cases of disease of the bones. Béraneck[12] has prepared a vaccine consisting of a mixture of two toxins with which he claims that during two years he has improved the condition of 60 per cent. of his cases numbering 90, of whom two-thirds were in the second or third stages, while Wilson[13] is also of opinion that this preparation is of value. Trevelyan,[14] however, is sceptical as to its advantages. Mahere[15] has isolated a bacillus closely resembling B. Mycoides, the injection of which is harmless to man. In some cases of phthisis this procedure has appeared to cure the disease, while good results have followed the local application of living cultures to old fistulæ, diseased bones, the middle ear and the pleural cavity. Dewar[16] has recently reported good results from the injection of an ethereal solution of iodoform intravenously. About five minims of a nearly saturated solution are used daily as a rule, but as much as 45 minims can be injected at once. Large injections, however, cause unpleasant symptoms and are apt to lead to thrombosis. Scognamiglio and Meyer[17] have treated 31 cases of phthisis, 10 of which were advanced, with glandulen, a preparation from the lymphatic glands of the sheep—an animal practically immune to tubercle. The drug, which is given in tabloid form, produced uniformly good results. Moorhead[18] reports on nine out-patient cases treated by intra-tracheal injections of izal. He considers that it has a good effect on the whole, but the injections must be frequently repeated. As much as 2 oz. may be given at a time. In rapidly advancing cases and where the larynx is affected it should not be used. Guerder[19] states that he has injected the active principle of cod-liver oil subcutaneously in 60 cases of tuberculosis; 40 patients were cured, 4 died, and the remainder were still under treatment. Landerer[20] claims good results from the injection of hetol, and Lardier[21] from the administration of yeast in doses of 5 to 20 grams daily. Burnet[22] has reported on 13 cases of phthisis treated by ichthyol for a period of some six months, eleven of which were benefited.

Dixon[23] notes that owing to the absence of vasomotor nerves to the branches of the pulmonary artery, pilocarpine, adrenalin, physostigmine, and digitalis have no effect on the pulmonary vessels, but that barium and calcium salts and veratrine, which act directly on muscle, can be used to check bleeding from the lungs.

[1] Lancet, Jan. 30, 1904, p. 278. [2] Amer. Jour. Med. Sci., Oct. 1903, p. 679. [3] Scot. M. and S. Jour., Jan. 1904, p. 440. [4] Med. Rec., Dec. 12, 1903, p. 959. [5] Ibid. [6] Ibid., p. 946. [7] Sem. Med., Dec. 2, 1903, p. 392. [8] Ibid. [9] Ibid. [10] Ibid. [11] Med. Rec., Dec. 12, 1903, p. 946. [12] Sem. Med., Dec. 2, 1903, p. 393. [13] Lancet, Jan. 16, 1904, p. 160. [14] Ibid. [15] Med. Red., Dec. 26, 1903, p. 1020. [16] Brit. Med. Jour., Nov. 21, 1903, p. 1328. [17] Allg. Med. Centralztng., No. 42, 1899. [18] Dubl. Jour. Med. Sci., Jan. 1904, p. 15. [19] Scot. M. and S. Jour., March 1904, p. 441. [20] Ibid. [21] Ibid. [22] Ibid. [23] Lancet, Jan. 16, 1904, p. 163.

